# New chimeric RNAs in acute myeloid leukemia

**DOI:** 10.12688/f1000research.11352.2

**Published:** 2017-12-19

**Authors:** Florence Rufflé, Jerome Audoux, Anthony Boureux, Sacha Beaumeunier, Jean-Baptiste Gaillard, Elias Bou Samra, Andre Megarbane, Bruno Cassinat, Christine Chomienne, Ronnie Alves, Sebastien Riquier, Nicolas Gilbert, Jean-Marc Lemaitre, Delphine Bacq-Daian, Anne Laure Bougé, Nicolas Philippe, Therese Commes

**Affiliations:** 1 Institut de Biologie Computationnelle, Université Montpellier, Montpellier, France; 2 Institut de Médecine Régénératrice et de Biothérapie, INSERM U1183, CHU Montpellier, Montpellier, France; 3 Laboratoire de Cytologie et Cytogénétique, CHU Caremeau, Nîmes, France; 4 Université Paris Sud, Université Paris-Saclay, Orsay, France; 5 Institut Curie, PSL Research University, Paris, France; 6 Institut Jérôme Lejeune, Paris, France; 7 Laboratoire de Biologie Cellulaire, Hôpital Saint-Louis, Assistance publique - Hôpitaux de Paris (AP-HP), Paris, France; 8 Hôpital Saint-Louis, Université Paris Diderot, INSERM UMRS 1131, Paris, France; 9 Instituto Tecnológico Vale, Nazaré, Belém, PA, Brazil; 10 CEA Institut de Génomique, Centre National de Génotypage, Evry, France

**Keywords:** chimeric RNA, RNAseq, acute myeloid leukemia, biomarkers, bioinformatics analysis, Complex Read Analysis, Classification (CRAC), PML-RARA, TRIM28

## Abstract

**Background:** High-throughput next generation sequencing (NGS) technologies enable the detection of biomarkers used for tumor classification, disease monitoring and cancer therapy. Whole-transcriptome analysis using RNA-seq is important, not only as a means of understanding the mechanisms responsible for complex diseases but also to efficiently identify novel genes/exons, splice isoforms, RNA editing, allele-specific mutations, differential gene expression and fusion-transcripts or chimeric RNA (chRNA).

**Methods:** We used Crac, a tool that uses genomic locations and local coverage to classify biological events and directly infer splice and chimeric junctions within a single read. Crac’s algorithm extracts transcriptional chimeric events irrespective of annotation with a high sensitivity, and CracTools was used to aggregate, annotate and filter the chRNA reads. The selected chRNA candidates were validated by real time PCR and sequencing.  In order to check the tumor specific expression of chRNA, we analyzed a publicly available dataset using a new tag search approach.

**Results:**  We present data related to acute myeloid leukemia (AML) RNA-seq analysis. We highlight novel biological cases of chRNA, in addition to previously well characterized leukemia chRNA. We have identified and validated 17 chRNAs among 3 AML patients: 10 from an AML patient with a translocation between chromosomes 15 and 17 (AML-t(15;17), 4  from patient with normal karyotype (AML-NK) 3 from a patient with chromosomal 16 inversion (AML-inv16). The new fusion transcripts can be classified into four groups according to the exon organization.

**Conclusions:**  All groups suggest complex but distinct synthesis mechanisms involving either collinear exons of different genes, non-collinear exons, or exons of different chromosomes. Finally, we check tumor-specific expression in a larger RNA-seq AML cohort and identify new AML biomarkers that could improve diagnosis and prognosis of AML.

## Introduction

High-throughput sequencing technologies (NGS) enable the detection of new biomarkers used for tumor classification and disease monitoring, including patient response to therapies. Whole-transcriptome analysis with RNA-seq is increasingly acquiring a key role, not only to learn about mechanisms responsible for complex disease, but also to identify novel genes/exons, splice isoforms, RNA editing, allele-specific mutation, differential gene expression, fusion-transcripts and chimeric RNA (chRNA) ^1, 2^.

For chimeric RNA, a group of fusion transcripts is increasingly used by geneticists in oncology diagnosis ^3, 4^. These cancer biomarkers are generated at DNA level from gene fusions by mechanisms such as translocations, inversions, or more complex chromosomal rearrangements. In some well-documented cases, gene fusions, in addition to contributing to neoplastic transformation, produce fusion RNA and proteins used as therapeutic targets (Mertens *et al.*, 2015 ^5^, Yoshihara *et al.*, 2015 ^6^ and references therein). However recent RNA-seq analyses have revealed the existence of an enlarged “chimeric transcriptome” ^5, 7– 10^ generated by new RNA processing events such as cis- and trans-splicing, whose mechanisms and functional roles are poorly understood. It is crucial to determine whether these events represent artefacts of RNA-sequencing, transcriptional noise with little impact on cell functions or tissue-specific transcripts, or whether these events are important in tumor development. Several significant examples of chRNA without corresponding fusions at DNA level have been described in the context of neoplasia ^11, 12^.

Both from a methodological and biological perspective, profiling chimeric RNA is a challenging issue. chRNA’s distinctive features must be identified to provide relevant biological information, including synthesis mechanisms and pertinence as a biomarker. Though RNA-Seq may enable new biomarker discovery, there is a lack of consensus on which analysis tool and algorithmic strategy should be used, especially in the detection of chRNA ^13^. We recently proposed Crac ^14^, a novel way of analyzing reads that combines genomic locations and local coverage to classify the biological events, to directly infer splice and chimeric junctions within a single read. The Crac software is based on an innovative algorithm that allows extraction of transcriptional events, irrespective of annotation. The main advantage of using Crac to detect chimeras is its high sensitivity and specificity, allowing detection of rare events with confidence. We developed the complementary CracTools module to aggregate, annotate, and filter the chRNA reads. The procedure classifies reads into 4 classes, depending on the exon organization. The first class corresponds to fusion transcripts arising from two different chromosomes, the second class includes parts of two genes belonging to the same chromosome strand. The third and fourth classes involve non-collinear transcription on the same chromosome. This categorization suggests that each class represents distinct, complex synthesis mechanisms.

In order to assess Crac’s potential to identify new biomarkers, we present data related to acute myeloid leukemia (AML) RNA-seq analysis. AML provides a practical cellular model to detect chRNA biomarkers in order to improve classification and patient follow-up in precision medicine ^2^. Despite the fact that leukemia chRNAs are well characterized, our study highlights new biological cases of chRNA. We identify and validate 17 chRNAs initially detected in 3 AML patients that belong to the 4 classes. We then explore their specific expression in the publicly available LEUCEGENE cohort ^15^, and propose new criteria for distinguishing chRNAs based on their recurrence, tumor, subgroup, or patient-specific expression. We also classify the chRNAs according to differences in expression of the genes linked to the chimera, in healthy donors vs AML patients. Finally, we identify new biomarkers that could improve diagnosis and prognosis of AML.

## Materials and methods

### AML samples and cell lines

Three sets of samples from patients with AML were used in this study. Each patient is designed by a specific ID including the OM or OS code corresponding respectively to bone marrow or blood sample ( Supplementary Table S1). The first set of 25 AML samples consisted of 11 AML-NK samples, 4 AML-inv16, 5 AML-UK, 2 AML-t(15; 17) also called Acute Promyelocytic Leukemia (APL), and 3 other AML-AK samples ( Table S1 lines 3 to 27). They were supplied by JBG (Biological Resource Center CHU-Nîmes, France) and included both RNA and peripheral blood mononuclear cells (PBMC), stored in RNALater (Ambion, USA) according to the manufacturer instructions. The second set of 14 AML samples consisted of 10 AML-UK, 2 AML-NK, and 2 AML AK. They were supplied by AM (Medical Genetic Unit, University St Joseph, Lebanon) ( Table S1 lines 28 to 41). They included blood and bone marrow stored in TRIzol reagent (Life Technologies, USA). The third set of AML samples included 3 AML-t(15;17), and were provided by CC and BC (Cell Biology Unit, Hôpital St-Louis, France). For the latter, peripheral blood mononuclear cells were collected by ficoll-hypaque density gradient and cultured at a concentration of 1×10 ^6^/ml, with or without 0.1µM ATRA, for 3 days. PBMC samples from healthy donors were pooled and used as control sample. The patients and healthy donors provided written informed consent to participate in the study, in accordance with the Declaration of Helsinki. The U937 leukemia cell line (DSMZ, Braunschweig, Germany), NB4 promyelocytic cell line and NB4-LR2 cell line (provided by CC) were cultured in RPMI 1640 (Invitrogen, USA) containing 10% decomplemented FBS (Dutscher, Brumath, France). For differentiation conditions, NB4 and U937 cells were cultured as previously described ^16, 17^. The chemical agents used for differentiation were 1µM all-trans retinoic acid (ATRA; Sigma-Aldrich, Gillingham, UK), or 0.1µM vitamin D (VD) associated with 500pg/ml transforming growth factor beta TGFβ (Promega Corporation, USA) for the NB4 cell line ^18^. For the U937 cell line, 0.1µM TTNPB associated with 1µM Targretin (LGD1069) and 0.1µM 1 alpha, 25 dihydroxyvitamin D3 (VD) were used. TTNPB, LGD1069 and VD were kindly provided by Dr Klaus (Hoffman-La Roche, Switzerland), JHiernaux (Glaxo-welcome Laboratories, France), and L Binderup (Leopharmaceutical products, Denmark), respectively. We also used human neuroblastoma cancer SH-SY5Y cells, human breast cancer MCF7 cells and human prostate cancer MDA-PCa cells. Cell pellets were kindly provided by S. Marchal (University Montpellier, France), and by D.Noel (Institute of Regenerative Medicine and Biotherapies, France) for the latter.

### RNA extraction and reverse transcription

RNA was extracted with the RNeasy Qiagen kit (Qiagen, Germany), additional DNase treatment was performed in order to remove residual DNA (RNAse free DNase set, Qiagen, Germany). Total RNA was quantified using a NanoDrop ^®^ ND-1000 spectrophotometer (NanoDrop ND-Thermo Fisher Scientific, USA). RNA quality and quantity were assessed using the 2100-Bioanalyzer (Agilent Technologies, Waldronn, Germany). Reverse transcription was performed with random primers and MultiScribe Reverse Transcriptase (High-capacity cDNA Archive kit; Applied Biosystems, USA), using 1 µg of total RNA. To check for possible chRNA formation induced by transcriptional artefacts, part of the samples were double reverse transcribed. In this case, the second reverse transcription reaction was performed with ImProm-II™ Reverse Transcriptase (Improm-II Reverse Transcription System, Promega, USA).

### RNA-seq experiments

Three AML samples (AML-NK, AML-t(15;17) and AML-inv16) taken from patients OM100011, OM110223 and OS110089, respectively (AML test group) from the Biological Resource Center, CHU-Nîmes, France, were selected for RNA-seq experiments. 4 µg of total RNA taken from bone marrow (OM100011, OM110223) or blood PBMCs (OS110089) were sent to GATC biotech and analyzed on an Illumina HiSeq 2000 to generate 100 base pairs of stranded RNA-Seq paired-end reads. The RNA-Seq was performed using polyA-selection with the truSeq RNA Lib-Prep Kit (Illumina, San Diego, CA) adjusted with GATC specific procedure for strand specificity. The following publicly available datasets of the LEUCEGENE project dedicated to Acute myeloid and lymphoid leukemia studies ^15^, have been used in this study (a detailed list is provided in Supplementary Table S6):

- GSE48846 (17 CD34 hematopoietic stem cell),
- GSE49642 part1 (43 AML-NK),
- GSE62190 part3 (82AML-AK with 24 inv16, 19 t(8;21),5 inv3,7 t(9;11), 5 t(6;11),6 t(11;19) and complex karyotypes)
- ENCODE publicly available datasets (Supplementary Table S7).

### RNA-seq analysis and chRNA extraction procedure using Crac and cracTools

RNA-seq analyses were performed serially using Crac (V2) and CracTools (V1.2) ^14^. Crac is a software for analyzing reads when a reference genome is available, that is completely independent of annotations. It ignores the sequence quality of reads and classifies reads by detecting diverse biological events (mutations, splice junctions, and chRNAs) and sequencing errors from a RNA-seq read collection. In this analysis, we used two Crac versions in succession (V1.6 and V2) to extract and classify chRNAs, with the GRCh37/hg19 genome as reference genome. Crac extracts the chimeric reads supporting the chimeric junction (spanning junction) made of a non-collinear arrangement of genomic regions ^14^. CracTools was then used to aggregate, annotate and filter the chRNA reads and extract the chimeric paired reads (spanning PE) ( Figure S1). Reads were annotated according to a GFF file from ENSEMBL Genome Browser (link to GFF file in Data Availability section) by giving priority to location in exons of the annotated genes. The GFF file was built from ENSEMBL (Ensembl 84 annotations). When reads were located on a non-annotated, transcribed region, the corresponding “NONE” annotation was mentioned. The procedure included classifying chRNA into four categories depending on exon organization, as described in the introduction. This classification resembles the one depicted in Gingeras, 2009 ^7^ and can be summarized as follows:

– Class 1, the exons are located on different chromosomes;
– Class 2, the exons are collinear but most likely belong to different genes, to be verified through the annotation;
– Class 3, the exons are on the same chromosome and same strand, but not in the order in which they are found on DNA;
– Class 4, the exons are on the same chromosome but on different strands.

For each analyzed chimera, the pipeline provides related information, including a unique read identifier annotated by the pipeline, class, number of spanning junctions and spanning reads:

1. ID: A unique read ID for each chimera, composed of ‘sample name: chimera ID’

2. Fusion gene names (left-right)

3. Chr(left): Chromosome number of the 5' part of the chimera

4. Pos1: Genomic position of the 5' part of the chimera

5. Strand1: Genomic strand of the 5' part of the chimera (+1 or -1).

6. Chr(right): Chr number of the 3' part of the chimera

7. Pos2: Genomic position of the 3' part of the chimera

8. Strand2: Genomic strand of the 3' part of the chimera

9. ChimValue: the chim-value takes into account methodological parameters and ambiguities, including the read mapping (P_loc) and the read coverage (P_support). ^13^

10. Spanning junction normalized: Spanning junction reads coverage (normalized per billion of reads). A spanning junction read is the read that contains the chimeric junction.

11. Spanning PE normalized: Coverage of paired-end reads (normalized per billion of reads) that contains the chimeric junction in the non-sequenced part

12. Class: Chimeric class from 1 to 4.

The filtering process with CracTools considered the following thresholds:

a. Only candidate fusions (chRNA) with at least one read covering the fusion breakpoint,
b. Spanning reads must be associated with pair-end reads,
c. An annotation of the fusion junction matching almost a known expressed sequence (gene A or Gene B),
d. A ChimValue of up to 60, allowing the removal of false positives corresponding to pseudogenes and splicing events detected by GSNAP.

Candidate fusion transcripts involving adjacent genes within a 3Kb distance region were discarded. To estimate the number of supporting reads for a chimeric candidate, CracTools extracted the count number of spanning junctions and spanning PE reads. All candidate fusion transcripts were validated using qPCR and Sanger sequencing except the class 3 overlap which requires systematic reconstruction of the fusion transcript for the design of primers. 

### Manual annotation

The potential chimeras are listed in Table S2 with the appropriate features. For each fusion transcript, the Crac software provided a reconstructed sequence comprising, on one hand, the chimeric junction sequence based on the most representative read, and on the other hand, the paired read sequence. The symbol (#) marks the segment of the read that was not sequenced and the (*) symbol marks the junction point ( Table S2 and Supplementary Figure S1). The sequences of reads, both junction and paired, which supported the chimera were mapped ( BLAT, UCSC) to the human genome GRCh37/hg19 in order to identify complex biological events (splicing, SNPs, insertions, deletions, repeats, polymorphisms, etc.). ****Complementary annotations were identified using the ENSEMBL genome browser to determine exons and spliced variants involved in the transcript. Protein sequences and functional annotations were also verified to identify affected protein domains and to evaluate potential protein damage in selected chRNAs.

### PCR validation

Reverse transcription was performed as described above. 1 µl of each cDNA sample (2ng/µl) was added to a 5 µl of reaction mix containing 3 µl of Master Mix (LightCycler ^®^480 sybr green I Master, Roche Diagnostics, GERMANY) and 0.66 µM forward and reverse primers. Mix and cDNA were loaded onto the 384-well PCR plate using an epMotion 5070 automated pipetting system (Eppendorf, Germany). Primer sequences were designed using the Primer3Plus web interface, with some constraints as described in the PCR strategy (see Supplementary Figure S1), and synthesized by Eurofins MWG Operon, Germany. The amplification area was centered on the junction, and primers were designed to tag each sides of the junction. Primers are listed in Supplementary Table S3. PCRs were carried out in 384-well plates on a LightCycler ^®^480 Real-Time PCR System (Roche Diagnostics, Germany). Amplifications were performed according to the following conditions: 95°C for 5 min, then 55 cycles as follows: 95°C for 10s, followed by T°C depending on Tm for 10s, and 72°C for 10s. Ultimately, a melting curve analysis ranging from 60°C to 95°C was performed to control primer specificity. For each sample, the graph of the negative first derivative of the melting curve gave a specific peak corresponding to the amplified transcript. Samples with TM value peaks different from those found in the negative control were considered potential positive targets and retained for sequencing. PCR products were purified with the Minelute PCR purification kit (Qiagen, Germany) and sequenced on the ABI 3730XL (Eurofins MWG Operon, Germany).

For the newly discovered Class1 chRNA, identified in patient OS110089 and corresponding to Chr2 and Chr13 positions and to PAN3-NONE annotations, the presence of chRNA was checked in leukemia samples obtained during the patient follow-up ( Figure S3). The NONE transcript expression was checked by qPCR in human embryonic stem cells HD129 (cDNA was kindly provided by J. De Vos, Institute of Regenerative Medicine and Biotherapies, France), in AML samples and in U937, NB4, SH-SY5Y, MDA-PCa and MCF7 cell lines. To this end, we designed forward and reverse primers on the 5’NONE sequence ( Figure S2).

### FISH Experiments

Molecular cytogenetics were performed on metaphases from bone marrow aspirate collected from the samples using a synchronised protocol. A first step aging slide with the cytogenetic preparation was performed by immerging slides in 2xSSC solution (saline sodium citrate) for 30 minutes at 37°C, followed by dehydration in 3 baths of increasing ethanol concentration: 70%, 85% and 100%, each for 1 minute. Finally, slides were air dried at room temperature. To confirm the putative t(2;13) translocation related to the PAN3-NONE fusion gene, a fusion probe was designed using bacterial artificial chromosome (BAC) technology, framing the following regions of interest: RP11-239J16, RP11-339H12 in chromosome 2p21 (labeled in Cy3 Orange) and RP11-179F17, RP11-95G6 in chromosome 13q12 (labeled in Alexa 488 Green) (BlueGnome, Cambridge, UK). 1 μl of each of the BACs was mixed and diluted in 9 μl of hybridization BlueFISH Buffer and then applied to the slide. Chromosomes fixed on the slide and probe of interest were denatured in a single step using Thermobrite (Abbot Molecular, USA). Codenaturation was done at 75°C for 5 minutes, followed by hybridization at 37°C in a humid atmosphere overnight. To remove the probe that would not properly hybridize, two successive washes in stringent conditions were performed: the first one in 0.4xSSC and 0.1% Igepal at 73°C for 2 minutes, the second one in 2xSSC and 0.3% Igepal for 1 minute at room temperature. After complete drying, 10 µl of counterstaining reagent (DAPI 125ng/ml, Abbott Molecular, Chicago, USA) was added. Slides were observed on an epifluorescence microscope (Olympus BX60). For both probes, positivity was defined as the presence of two fusion signals (co-location) in addition to a red signal and a green signal.

### Tag search approach and gene expression clustering

The tag search approach consisted of extracting representative sequences (Tags) 30 nucleotides in length, centered on the chimeric junctions. The latter were then searched in LEUCEGENE and ENCODE publicly available RNA-seq datasets. A FASTA file, listing these tags of interest, designed for each chRNA, was submitted to a specific pipeline (countTags, https://github.com/jaudoux/countTags) that searched for sequences and their reverse complement with an exact match in the FASTQ files. For each FASTQ file and each tag, the total number of tags (total k-mers) is counted. Final value is given in a delimited table, with a tag count normalized per 5 billion of k-mers.

For the gene expression clustering, chRNA were selected as below. Chimeric genes were extracted using cracTools predictions for samples OS110089, OM100011 and OM110223. Among all chimeric junctions detected, only those having a "chimValue" greater than 75 and with at least 3 spanning reads were conserved. Read-through chimeras were further selected based on three criteria:

i. Chimera annotated as "Class 2" (collinear transcription),
ii. Short fusion distance (max 300kb),
iii. Short exon-end distance (max 20bp).

Tandem repeat chimeras were further selected based on three criteria:

i. Chimera annotated as "Class 3"
ii. Overlaping chimeric fragments (the two parts of the chimeric read correspond to overlapping sequences on the genome)
iii. Both chimeric fragments are located on the same exon of the same gene.

For each chimera type (read-through and tandem-repeat), only genes involved in at least 2 different chimeric events (either from the same or different samples) were finally selected as candidates for the clustering of gene expression. LEUCEGENE data were downloaded from SRA using the fastq-dump utility (version 2.5.4) and converted to FASTQ. Using Kallisto 0.42.4 software and Ensembl 84 annotations, we determined transcript expression. Transcript counts computed by Kallisto were merged at gene-level ^19^. The normalization of counts was performed with DESeq2 (version 1.14.1) (design ~ 1), so values used in the clustering were normalized counts transformed with the variance stabilization method provided in DESeq2 package. Heatmaps were produced with heatmap.2 function from the gplots package (R version number 3.3.2), using default parameters (i.e. complete-linkage clustering and Euclidean distance).

## Results

### Validation of chRNA candidates

We performed RNA-seq on samples from 3 AML patients. One presented with a normal karyotype (NK), while the other two presented with an abnormal karyotype (AK), one with an Inv16 and the other with a t(15,17) translocation (sample names 1–3, Supplementary Table S1). The sequences were analyzed using Crac and CracTools. The selected chRNA candidates were tested by qPCR, and sequenced when qPCR displayed a positive signal. Some candidates could not be validated by qPCR due to the difficulty in designing suitable primers. The CBFB-MYH11 and PML-RARA fusion transcripts expressed in the AML-inv16 and AML-t(15;17) samples were identified using both RNA-seq and qPCR analysis, confirming the reliability of RNA-seq and the Crac suite in this type of analysis.

We identified 17 chRNAs among 44 candidates; 10/23 from AML-t(15,17), 4/18 from AML-NK, and 3/3 from AML-inv16 ( Supplementary Table S2 and Supplementary Table S4). The validated chRNAs were distributed into 4 classes (definitions of these are in the materials and methods section) as follows:

– Class 1: 5
– Class 2: 3
– Class 3: 5
– Class 4: 4.

Among the Class 1 chRNAs more frequently associated with genomic translocation, we identified 4 chRNAs associated with PML and RARA genes in patient OM110223 suffering from AML-t(15,17). The remaining chRNA of this class was associated with the PAN3 gene and a non-annotated region (NONE), and was found in patient OS110089 suffering from AML-inv16. We highlighted two types of Class 2 chRNAs that depend on the genomic distance between the two parts of the read. A short distance was consistent with read-through, whereas a long distance would be associated with other mechanisms. We found two kinds of Class 3 chRNA - in the first, the chRNA processes the 3’ exon before the 5’ start of the same gene. In the second, the chimera involve distant exons from different genes. Finally we also validated several Class 4 chRNAs, among which the CBFB-MYH11 chimera associated with Inv16.

### New Class 1 PAN3-NONE chRNAs associated with a genomic translocation

**Figure 1.**
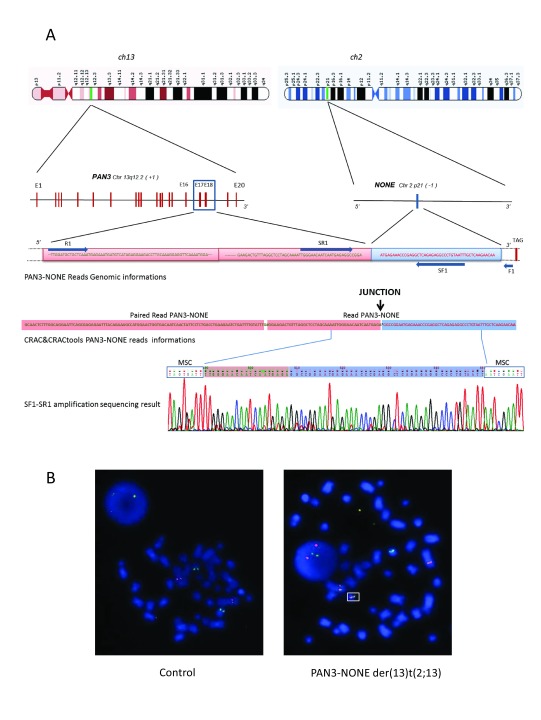
A new specific Class 1 PAN3-NONE chimera. **A**) Fusion junction description and primer design. The sequences of spanning junctions and paired reads supporting the chRNA and the corresponding designed primers are indicated. The orange and blue squares represent respectively the E17 and E18 of PAN3 gene (E16 of ENST00000399613.1 transcript), and the NONE part of the fusion transcript. The sequencing result of the PAN3-NONE SF1-SR1 PCR product is indicated. The symbol (#) denotes the part of the read which was not sequenced, (*) denotes the junction, and MCS indicates the multiple cloning sites used to clone the PCR product. ( **B**) Translocation checking by FISH. The left panel (Control) represents normal blood cells and the right panel represents cells from patient OS110089. The presence of the NONE sequence on chr2 is denoted by the red signal and the presence of the PAN3 sequence on chr13 mentioned by the green signal. The right panel presents the NONE-PAN3 fusion at the chromosomal level (white box).

A new Class 1 chRNA was identified in patient OS110089 presenting the inv16 associated fusion transcript CBFB-MYH11. The new fusion junction read (n° 14888 PAN3-NONE; Supplementary Table S2) corresponds to 13q12.2 and 2p21 chromosomal locations at the 5’ and 3’ of the RNA, respectively. The read chimeric annotation indicates a fusion between the 3’end of PAN3 exon18 (chr13+), with a non-annotated transcribed region “NONE” (chr2–pos 43193199-43193247) as shown in Figure 1A. The PAN3-NONE fusion transcript was validated by qPCR, and was only detected in patient OS110089 as shown in results of the AML cohort ( Figure 5A). We compared its qPCR Ct value with the Ct value obtained with the amplification of the CBFB-MYH11 transcripts, and noticed that its value was higher (32 cycles vs 28, Supplementary Figure S3). This difference could be correlated either to a lower transcript expression level or to a heterogeneous expression in the tumor cell population. In order to determine whether this fusion transcript is associated with chromosomal rearrangement, FISH experiments were performed with a custom fusion probe. A corresponding translocation was observed in only 31% of the leukemia cells, demonstrating that the PAN3-NONE transcript belongs to a subclone, which could explain the lower expression level observed ( Figure 1B). The analysis of the PAN3-NONE transcript during patient follow-up revealed its disappearance after the first induction of chemotherapy, without reappearance during the relapse period ( Figure S3).

**Figure 2.**
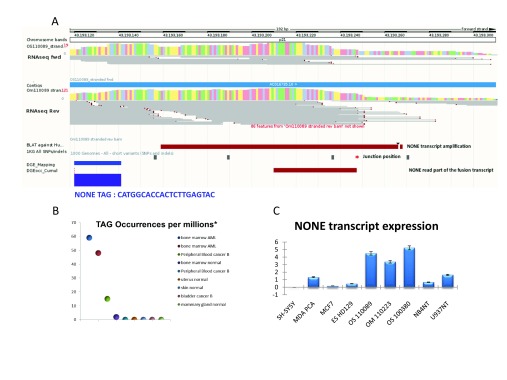
NONE transcript sequencing and expression. **A**) Display of the ENSEMBL genome browser viewer for the NONE transcribed region. The blue horizontal bars represent the genomic sequence. The histogram of the RNA-Seq coverage in the chromosomal region is displayed on both strands (OS110089 stranded and RNA-seq fwd/rev tracks). Public and personal DGE data (‘DGE tag location’ track: blue rectangle for occurrence>2) are displayed on both strands of the chromosome, with their relative occurrences (histogram of ‘DGE expression level’ track) using a private DAS server. ( **B**) Relative expression of the NONE tag per million reads in the DGE datasets (Philippe, *et al*. 2013). ( **C**) Relative expression of the NONE transcript in different cell lines and AML samples (NT refers to not treated cells). The relative gene expression was determined using the 2-ΔΔCT method. Transcriptional modulation was calculated by comparing various lineages with SH-SY5Y (subline of the neuroblastoma cell line SK-N-SH). For normalization, RPS19 was selected as a reference transcript. Standard deviation was measured using duplicate.

We next investigated the transcription of the non-annotated region “NONE” (chr2–pos 43193199-43193247) in normal and tumor tissues using the approach described previously combining digital Gene expression (DGE) and RNA-seq data ^20^. Querying tissue expression profiles with DGE tags, we observed a tag in the NONE chromosomal area showing a specific expression in AML samples ( Figure 2B). The RNA-seq read coverage and the DGE tag ( Figure 2A) confirmed a new transcribed region, validated by qPCR in tumor cell lines ( Figure 2C). We confirmed the presence of the NONE specific expression in AML and normal CD34+ hematopoietic stem cells (HSC) using a tag search approach in a largest RNA-seq collection of normal and tumor tissues ( Table S5 and data not shown). Together, these results revealed a new lincRNA specifically expressed in hematopoietic lineage and most highly expressed in AML samples. Moreover, this lincRNA contributed to the formation of a new chRNA in the case of a translocation between chromosome 13 and 2.

### New Class 1 PML-RARA variants

Four Class 1 junctions involving PML and RARA genes were identified from the analysis of the AML-t(15; 17) OM110223 sample ( Supplementary Table S2). Acute promyelocytic leukemia (APL) molecular diagnosis and minimal residual disease (MRD) monitoring are currently based on bcr1, bcr2 or bcr3 fusion transcript detection, depending on the DNA breakpoint ^21, 22^. PCR and FISH analyses performed during diagnosis showed that patient OM110223’s leukemia cells present a short bcr3 transcript which connects exon 3 of the PML gene and exon 9 of the RARA gene (equivalent to the exon 3 of the RARA-001 ENSEMBL transcript).

**Figure 3.**
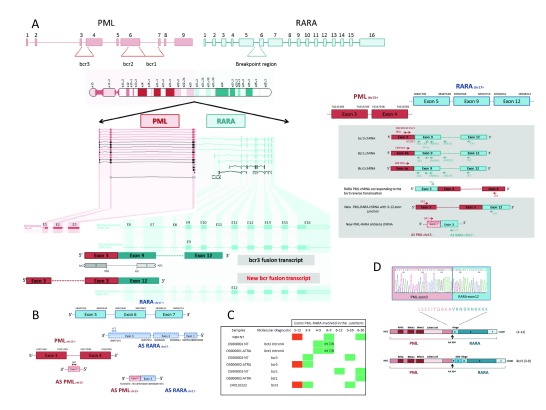
Identification of new PML-RARA fusion transcripts. ( **A**) Scheme of PML and RARA genes are shown on the top, with the potential breakpoints. Below is the schematic of the chromosomal translocation, with visualization of the short bcr3 fusion transcript. Gene structures for both PML and RARA genes (ENSEMBL genome browser), and exons involved in new PML-RARA chimera are shown. We assigned a number to exons ( **E**) of each gene. Bcr3 fusions found in this study are shown below, including the newly found fusion between exon 3 of PML and exon 12 of RARA. ( **B**) New RARA-PML antisense fusion transcript. Schematic of RARA (blue) and PML (red) antisense transcript positions relative to their sense transcript, and the resulting antisense fusion transcript, are shown below. ( **C**) Identification of selected PML-RARA transcript junctions in acute promyelocytic leukemia samples and NB4 cells by qPCR. ( **D**) Hypothetical incidence of PML-RARA fusion transcript on protein domains. Sanger sequencing results of the 3-12 PML-RARA fusion is shown on the top. The hypothetical protein product is presented below, as well as the protein from the exon 3–9 fusion.

Among the 4 PML-RARA characterized junctions, one of them confirms the short bcr3 variant expression and the “reciprocal” transcript joining the RARA exon 5 and PML exon 4 ^23^. Besides these fusion transcripts already described ^23, 24^, we observed the presence of two new fusion transcripts. The first corresponds to a fusion RNA shorter than bcr3, joining PML exon3 and RARA exon 12 ( Figure 3A). This junction was detected in NB4 cells which express a bcr1 transcript as well as in ATRA treated cells from another bcr3 patient (OS000002 in Figure 3C). The corresponding protein lacks the RARA DNA binding domain present in previously described fusion proteins ( Figure 3D). Moreover, different fusion transcripts linked to the translocation between the chr15 and 17 can coexist in a tumor sample, with their expression changing with time or treatment ( Figure 3C). The second new fusion transcript corresponds to a chRNA that joined the exon 3 of a known RARA antisense transcript with the antisense part of PML intronic region ( Figure 3B). The primers were designed in the corresponding PML intron free from known transcription, in order to amplify the antisense chRNA. This transcript was only detected in the sequenced sample. The sequencing results of PML-RARA chRNA qPCR amplifications revealed another fusion transcript joining PML exon 3 and RARA exon 10 present in OM110223 leukemia cells, without corresponding spanning junction read.

### New Class 2, 3 and 4 chRNA

**Figure 4.**
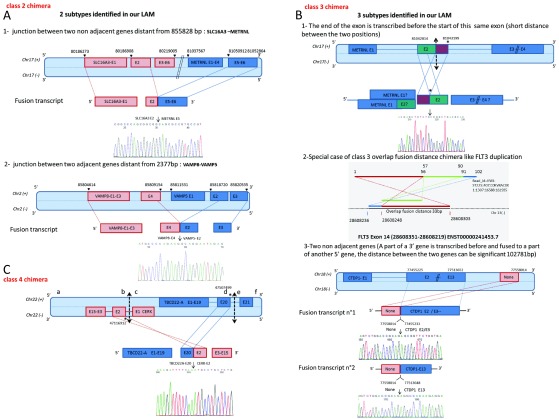
Different classes of validated chRNA. The position and orientation of the genes on the chromosomes have been schematically arranged. The start and end positions of the exons have been indicated. The representations of the chRNA junction supported by the Sanger sequencing results are also shown. ( **A**) Two subtypes illustrating Class 2 chimera junctions. The first subtype ^1^ is illustrated by the junction of two non-adjacent genes SLC16A3 and METRNL, separated by 855828 bp. The second subtype ^2^ is illustrated by the junction between adjacent genes VAMP8 and VAMP5, separated by 2377 bp. ( **B**) Three subtypes illustrating Class 3 chimera junction. They all involve the inversion of exon order, first two concern an inversion within a same exon of a gene (METRNL and FLT3 cases), and the third subtype involves an inversion of a transcribed sequence, separated by about 103 000 bp (NONE-CTDP1). ( **C**) A Class 4 chimeric construction joining an exon from (+) strand gene TBCD22A with an exon from (-) strand gene CERK, arising from the same chromosome 22.

Four classes of chimera were identified using the Crac and CracTools pipeline. Associated with specific features and annotations, subcategories could be defined and linked to potential biological mechanisms ( Figure 4). Among the Class 2 chimera, we observed two distinct categories, depending on the vicinity of the two relevant genes ( Figure 4A). The first category involves a junction between non-adjacent exons separated by thousands of base pairs (SLC16A3-METRNL and UBR5-AZIN1, Figure 4A and Table S2). The validated fusion transcript joins the SLC16A3 exon 2 with the METRNL start exon 5 at a distance of 855828bp. As shown from RNA-seq data, the region between the two genes is transcribed (data not shown), which calls into question the hypothesis of an intra-chromosomal deletion.

The second subcategory concerns two adjacent genes, fused with a loss of some exons. In the illustrated example of VAMP8-VAMP5, VAMP8-001 transcript exon 4 joined VAMP5-001 transcript exon 2 ( Figure 4A). The subgroup most likely defines a read-through transcript category with an alternative splicing.

The Class 3 chimera could reflect three different mechanisms associated with: genomic duplication, polymorphism, or intrinsic transcriptional mechanisms ( Figure 4B). Two cases concern intra-exonic transcripts, with one exon end being present before the start of the same exon. This subcategory is illustrated by two fusion transcripts (METRNL; Figure 4B and RNF220 data not shown). In the transcript METRNL-001, the 3’ part of exon 2 (chr17 (+)/pos 81043015-81043199) is present upstream of the 5’ part of exon 2 (chr17 (+)/pos 81042813-81042850). Such transcript could be generated either by transcriptional event or by the transcription of a rearranged allele. Indeed, this subgroup, intragenic chRNA, could highlight the presence of tandem duplication. As an example, we also identified the well-known FLT3 tandem repeat involved in acute myeloid leukemia. This type of tandem duplication is characterized by an overlap in the read’s genomic positioning, which can be easily detected using our workflow ( Figure 4B).

The last case involves two distant genes. The genomic location of both transcripts is on the same chromosome but at a distance of 102781bp. We identified the chimera NONE-CTDP1 in the OM110223 patient sample. The transcript of a non-annotated chr18 (+)/pos 77557943-77558014 segment is fused with exon2-CTDP1-001 or with exon13-CTDP1-001 ( Figure 4B).

The Class 4 chRNA corresponds to reads whose 5’ and 3’ parts match on the chromosome’s opposite strands ( Figure 4C). This kind of chimera reflects a genomic inversion like CBFB-MYH11 in the absence of overlapping elements in the read. Besides the widely described inv16 (CBFB-MYH11), we validated three chRNA linked with a possible chromosomal inversion (TBC1D22A-CERK, MAEA-CTBP1, DHRS7B-TMEM11). In the TBCD22A-CERK fusion transcript, the TBCD22A exon 20 end is fused with the CERK exon 2 start ( Figure 4C).

### Recurrence of chRNA in normal hematopoietic stem cells and AML

**Figure 5.**
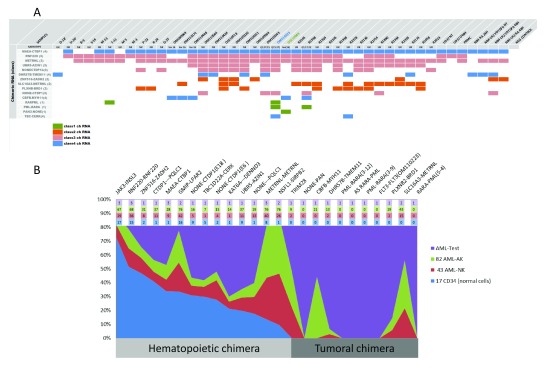
Chimeric RNA recurrence screening. ( **A**) Expression of validated chRNA in a large AML cohort. ChRNA classes are identified by colours (green for Class 1, orange for Class 2, red for Class 3 and blue for Class 4). Karyotypes are indicated below the sample name (UK for unknown karyotype, NK for normal karyotype). For abnormal karyotype, chromosomal rearrangement is indicated. The screening was also performed on not treated (NT) or differentiated cell lines. For PML-RARA and CTDP1 exons involved in the chRNA are indicated. ( **B**) Classification of chRNA depends on normal or tumor level expression. ChRNA expression, in normal CD34+ HSCs (CD34) and in AML (LEUCEGENE data), is presented using the tag search approach. For each group (AML-test, AML-AK, AML-NK, CD34), samples displaying a positive chRNA tag count are selected. The average tag expression is calculated with the selected samples and chRNA classified by their relative expression in the CD34 group. The table with highlighted colour indicates for each chimera, within the different cohort, the number of samples presenting the TAG.

To extend our analysis, we tested the expression of the validated chRNA in a large cohort of AML samples with different karyotypes ( Figure 5A, Table S1) using qPCR. We then classified the chRNA into AML subtypes or tumor-specific chRNA subgroups, taking into account the frequency and tissue-specific expression. We also distinguished the non-tumor chRNA by examining their expression in normal PBMCs. Among the 15 chRNA tested, some of them were widely expressed in all AML subtypes (RNF220-RNF220, METRNL-METRNL and MEA-CTDP1). The frequency and tissue-specific expression did not depend on the chRNA classes.

In order to validate our strategy on a large cohort, we analyzed a publicly available dataset of 125 AML and 17 normal CD34+ HSC RNA-seq using a tag search approach (see Materials and methods). For this purpose, we selected qPCR validated chRNA ( Table S4) and candidates previously untested due to difficulty in designing primers ( Table S2). The chRNAs were classified by their relative expression in normal CD34+ HSCs, in order to distinguish non-tumor and tumor-specific ones ( Figure 5B). Among the chRNAs expressed in CD34+ HSCs, we observed different profiles: those more highly expressed in normal CD34+ cells than in AML, and those with low expression in CD34+ HSCs (see NSFL1-SIRPB2, METRNL-METRNL).

We identified four new types of tumor-specific chRNA; TRIM28-TRIM28, DHRS7B-TMEM11, PLXNB-BLRD1 and SLC16A3-METRNL, expressed in all AML groups ( Figure 5B). DHRS7B-TMEM11 and PLXNB-BLRD1 transcripts are most abundant in the AML test group, whereas TRIM28-TRIM28 and SLC16A3-METRNL are equally expressed in the three AML groups (AML-test, 82 AML-AK, 43 AML-NK). It is worth noticing the involvement of the METRNL gene in two identified chimeras. TRIM28-TRIM28, FLT3-FLT3, PML-RARA (with (3–9) or (5–4) junction) and CBFB-MYH11 tag counts showed high mean expression levels (see Figure S4). FLT3, PML-RARA and CBFB-MYH11 are strong markers in AML, and also useful for prognostic and MRD monitoring. The FLT3 tag identified in OM110223 is not found in other samples, yet the pipeline reveals other FLT3 fusion transcripts, with different sequences in other AMLs (data not shown), indicating several variations at this fusion point. Among the new, TRIM28-TRIM28 chRNA is present at low frequency in normal and abnormal AML karyotype (2/43 AML-NK and 9/82 AML-AK). The high expression level of this chRNA in positive samples (comparable to previously described known markers CBFB-MYH11, FLT3-FLT3 and PML-RARA), suggests it could have a key role in such tumors.

**Figure 6.**
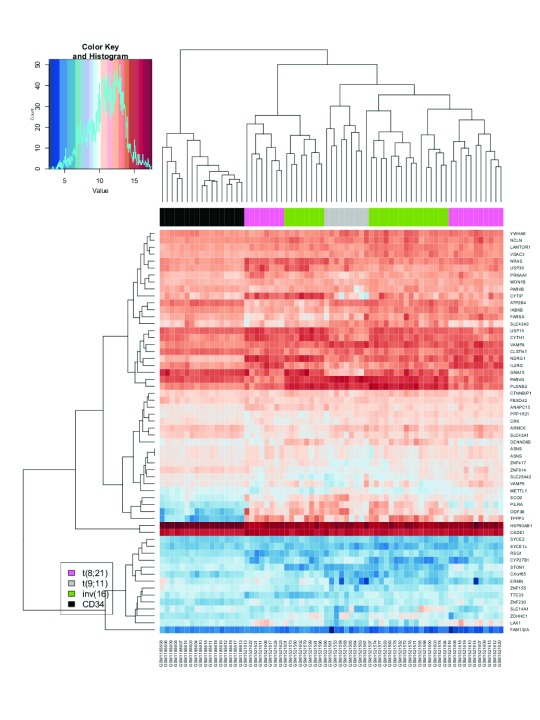
Expression profile of genes involved in read-through Class 2 chRNAs. The gene expression profile is analyzed by an unsupervised clustering method. Normal CD34+ HSC (CD34) and AML subgroups (LEUCEGENE RNA-seq data) were compared by analyzing the expression of a set of genes involved in read-through chRNAs. Parameters for gene selection are described in the Materials and methods section. Samples are identified by colours (black for normal CD34, pink for AML t(8;21), grey for AML t(9;11), green for AML-inv16). Gene names are indicated on the right.

In order to verify whether genes involved in chRNA have an aberrant expression profile ^25^ that could influence tumorigenesis, we analyzed the impact of gene expression on AML with unsupervised clustering. As described for the read-through chRNA subcategory, we compared RNA-seq data from normal CD34+ HSC and AML-AK subtypes (LEUCEGENE, part3). Figure 6 shows a quantitative analysis obtained with “read-through related” genes of the input cohort. Known AK-AML subgroups could be distinguished from CD34+ HSC by their expression profile. We performed a similar study with the Class 3 tandem duplication subgroup, showing interesting differential profiles with “tandem duplication related” genes, mostly comprised of the newly identified TRIM28, CEBPD and FLT3 ( Supplementary Figure S5).

## Discussion

The use of RNA-seq to provide a detailed view of the transcriptome and to detect new RNA transcripts, opens up new opportunities for improving diagnosis and treatment of human diseases. The characterization of new chRNA presents as a great opportunity, as it could reveal new transcriptomic biomarkers for cancer and therefore could be useful in personalized medicine. ChRNAs, also known as “fusion RNA” or “canonical chimeras” ^5, 26^, are already used in diagnosis, but many other chimeric fusion products generated by transcriptional mechanisms such as read-throughs, cis or trans-splicing ^5, 7, 9^, also have the potential to be used in diagnosis if correctly categorized. In this study, we successfully developed new tools to classify these fusion transcripts methodologically and biologically into chRNA categories and subcategories, and associated them with biological mechanisms.

One of the major challenges in chRNA detection is to distinguish true candidates from false positives during RNA-seq analysis. False positives can result from technical artefacts that occur during sample preparation, mainly produced by reverse transcription or downstream PCR errors ^27^. Bioinformatics processes also generate artefacts associated with the algorithm’s approach for mapping raw reads to the complex reference genome. Many attempts have been made to improve the bioinformatics analysis of chRNAs by proposing multistep filtering pipelines including gene annotation, lists of known fusion genes or machine learning approaches to improve prediction ^13^. However, pipeline choice remains a difficult task for biologists and bioinformaticians. We have developed a benchmarking system that enables the calibration and selection of pipelines optimised for the detection of fusion RNAs. Our work also entailed developing, within Crac, a machine learning model used to optimise the selection of fusion RNAs (personal data, submitted for publication). Crac offers precise prediction of all chRNA categories, and the CracTools pipeline helps the biologist increase biological rate validation, producing a ChimValue that takes into account methodology and annotation.

As mentioned above, what is at stake is the possibility of exploring the “whole chimeric transcriptome” to classify chRNA, and hence identify cancer biomarkers. High throughput studies, like the Cancer Genome Project ^6, 28, 29^, performed on large cohorts with thousands of experiments, have focused on “canonical fusion genes”. Most of the molecular cancer markers are based on fusion genes because of their ease of detection. It is more difficult to identify features that distinguish chRNAs into new categories, and to elucidate their biological mechanisms and functions. Integrating DNA-seq and RNA-seq data to improve classification is a possible solution ^5, 30^, but it is time and cost consuming. In this work, we confirm that RNA-seq is a good solution for canonical and new chRNA extraction, and propose a classification system based on subcategories and specific expression profiles.

The present study also reveals new chRNA candidates among the well characterized subcategories. We identified a Class 1 PAN3-NONE chRNA transcript associated with a new translocation in a tumor subclone of a characterized Inv(16) AML, that could be used in patient follow-up. We also identified novel PML-RARA isoforms, shorter than the isoforms currently used in diagnosis, which could again be used in patient follow-up. A recent publication revealed that several isoforms can coexist in leukemia cells from the same patient. The authors showed that the ATRA cell response is isoform-dependent, as the short isoform lacks sensitivity to ATRA ^31^. MRD and patient follow-up in APL is usually performed by PML-RARA transcript QPCR, and relapse is associated with an increase of bcr1, bcr2 or bcr3 fusion transcripts ^32^. Then, it would be useful to have a picture of the complete isoforms, to best address treatment in this context. Though many patients with AML-inv16 or AML-t(15;17) can benefit from effective treatment, some may develop resistance, leading to adverse outcomes. The appearance or increase of fusion transcripts during treatment could be an indicator of such resistance.

Besides the translocation and inversion mechanisms, our pipeline highlights other events that correlate with chromosomal rearrangement and cancer diagnosis and prognosis. We find Class 3 overlap fusion transcripts like FLT3, corresponding to tandem duplication that could be of use in the prognosis and MRD of AML ^33^. The real advantages of RNA-seq in highlighting tandem repeat sequences are its open nature and its capacity to detect outside “hotspots”. Furthermore, we also detected fusion transcripts resulting from “read-through transcription”, described in chRNA studies on cancer.

Most newly identified chRNAs used canonical splice sites and were detected in normal hematopoietic tissues. This observation confirms previously published works concerning the recurrence of chimeric fusion RNAs in healthy cells ^34^. It is unlikely that they are the products of genomic abnormalities, since they are expressed in healthy samples. However, the pathogenetic impact of these chimeric fusions remains unclear. Recent findings have demonstrated the role of read-through chRNA in renal carcinoma and breast cancer ^26, 35^, and our data demonstrates that genes involved in these events are differently expressed in AML. More studies are needed to elucidate the physiopathological impact of these chRNAs.

The potential of NGS technologies, particularly of RNA-seq, in increasing the capabilities of personalized medicine is clear ^2^. However, to achieve this, efforts must be made to facilitate the interpretation of complex high throughput data. For chRNA, this is feasible only if the fusion transcripts are well classified and characterized. New technologies are available to simplify disease follow-up at reduced costs. Here, we propose a robust, open method based on a single process to identify different classes of chRNA. This approach provides a chRNA transcriptome map of biomarkers for disease characterization and monitoring including known canonical gene fusions and new chRNA. In combination with a tag-based approach and gene expression profile, this map can give a global picture of the complex physiological processes and could correlate with current leukemia classification.

**Abbreviations:** chRNA, chimeric RNA; AML, acute myeloid leukemia; NK, normal karyotype; UK, unknown karyotype; AK, abnormal karyotype; APL, Acute Promyelocytic Leukemia; PBMCs, peripheral blood mononuclear cells; Inv16, chromosome 16 inversion; t(15;17), translocation of chromosomes 15 and 17; qPCR, quantitative polymerase chain reaction; NONE, non-annotated region; LincRNA, Long intergenic noncoding RNAs; Bcr, break chromosomal region; FISH, Fluorescence *in situ* hybridization; ATRA, all-trans retinoic acid; VD, vitamin D; TGFβ, transforming growth factor beta; MRD, minimal residual disease.

## Data availability

The data referenced by this article are under copyright with the following copyright statement: Copyright: © 2017 Rufflé F et al.

Data associated with the article are available under the terms of the Creative Commons Zero "No rights reserved" data waiver (CC0 1.0 Public domain dedication). 

The Crac and CracTools software is hosted on http://crac.gforge.inria.fr/.

Raw data (FASTQ files) for OM100011, OM110223 and OS110089 patients are available under accession number E-MTAB-5767 in the ArrayExpress database at EBI.

The publicly available ENCODE datasets (a detailed list is provided in Supplementary Table S7) used in the study are available on https://www.ENCODEproject.org/.

The following LEUCEGENE datasets: GSE62190 (82AML-AK), GSE48846 (17 CD34), GSE49642 (43AML-NK) (a detailed list is provided in Supplementary Table S6) are available on https://www.ncbi.nlm.nih.gov/geo/

 GRCh37/Hg19 genome sequences are available at ftp://ftp.ensembl.org/pub/grch37/release-88/fasta/homo_sapiens/dna/.

The annotations GFF file from the ENSEMBL genome browser is available at ftp://ftp.ensembl.org/pub/grch37/release-84/gff3/homo_sapiens/Homo_sapiens.GRCh37.82.gff3.gz.

## Consent

The patients and healthy donors provided written informed consent to participate in the study, in accordance with the Declaration of Helsinki. The study was approved by the ethics board of Nîmes University (CCPPRB 2002/1103).

## Supplementary material

**Figure S1: PCR strategy**

PCR primers were designed on both sides of the junction point (*) using spanning junction read or a sequence reconstructed by combining paired-end and chimeric spanning reads (meta-read). SF and SR indicated respectively short forward and short reverse primers, LF and LR, long forward and long reverse primers. Forward (F) and reverse (R) indicated primers pairs designed on spanning meta-reads.

**Figure S2: NONE transcript amplification strategy**

The PAN3-NONE read sequence and its corresponding genomic position on GrCH37 is shown. The junction point indicated the chr2: 43193247 (-) positions for NONE transcript. From this position, we selected an upstream 100 base pairs sequence in order to amplify the putative NONE transcript. Primers were designed on each part of the chr2: 43193247 position to specifically amplify the non-chimeric transcript.

**Figure S3: PAN3-NONE qPCR amplification**

Both PAN3-NONE and CBFB-MYH11 chRNAs were amplified from each sample. CBFB-MYH11 chRNA, was used as a positive control. For each amplicon, the Ct value and the melting temperature (Tm) are shown (upper table).

The second table shows PAN3-NONE and CBFB-MYH11 qPCR results for the time points corresponding to the 2 years follow-up of the OS110089 patient. Clinical information and QPRC results for both fusion transcripts are mentioned. The molecular biology for CBFB-MYH11 (Inv16) performed by the diagnostic laboratory is also indicated as positive or percentage values; DNQ= detected-not quantified; ND= not detected.

**Figure S4: Expression of ChRNA in different AML groups and hematopoietic cells by tag counting approach.**

For each group (AML-test, AML-AK, AML-NK and CD34), the average Tags mean expression is indicated. Tags were designed for the indicated chRNA as described in materials and methods. Only positive samples were retained to calculate the average tag expression. The tag counts are normalized per 5 billion of total k-mers.

**Figure S5: Expression profile of genes involved in Class3 tandem repeat chRNAs.**

Heat map representing colour-coded expression levels of class3 chimeras (chRNAs normalized count) across patient samples. Names of genes involved in class3 chRNAs are indicated on the right. Black, pink, green and grey bars at the top represent respectively normal CD34, AML-t(8;21), AML-inv16, AML-t(9;11).

**Table S1: Patient data.**

ID and patient information for the AML cohort.

**Table S2: CRAC & Cractools pipeline output obtained from OS110089, OM100011 and OM110223 RNAseq data analysis.**

**Table S3: Primers table for chRNA qPCR validation.**

**Table S4: Validated chRNAs information**

The table gives the list of TAGs used for tag search approach. For the chRNA, exons involved in the chimeric junction and tag sequences used for the tag search approach are indicated.

**Table S5: NONE and PAN3 tags expression within a set of libraries**

Specific tags for PAN3 and non-chimeric NONE transcript corresponding respectively to Chr13(+) : 28813770-28813799 and Chr2(-) : 43193287-43193316 position on GRCh37/Hg19 genome were designed and counted in FASTQ files of 52 libraries from various tissues. Expression was normalized per 5 billion of total k-mers.

**Table S6: List of LEUCEGENE and ENCODE datasets used in the study**

Table S6 describes the different parts of the LEUCEGENE cohort used in the study. Samples indicated by a GSM number are classified by groups of different Karyoptype. Table S6 also indicates the ENCODE dataset used in the study.
