# Supplementary figures and images for: New chimeric RNAs in acute myeloid leukemia
Source: F1000Res. 2017 Dec 19;6:ISCB Comm J-1302. Originally published 2017 Aug 2. [Version 2] doi: 10.12688/f1000research.11352.2 (PMC5861515; doi:10.12688/f1000research.11352.2)

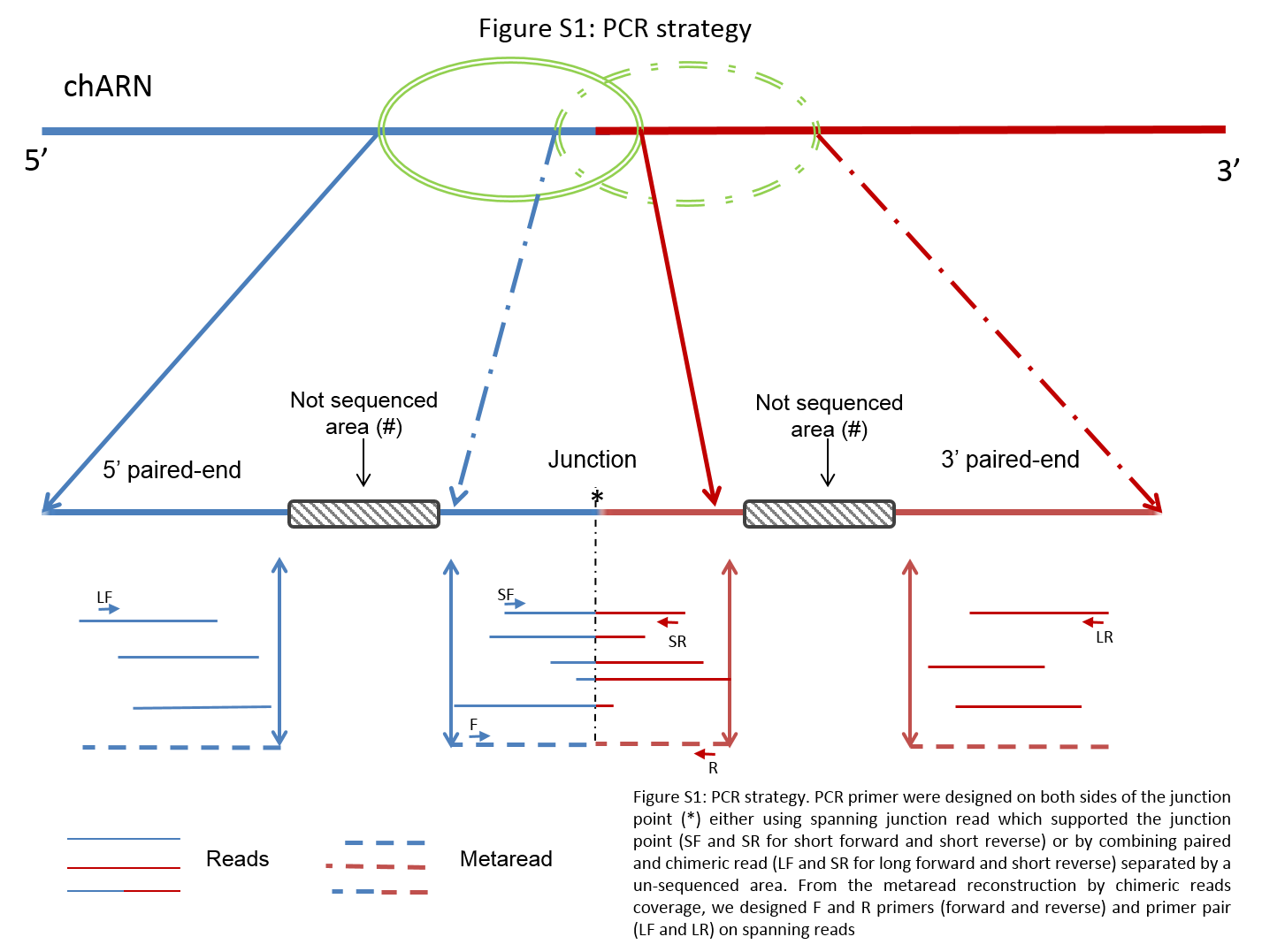

Supplement: Supplementary file 1 [file f1000research-6-14532-s0000.tgz › 351f2910-409f-47f7-a0ab-658f751f75e0.tif]

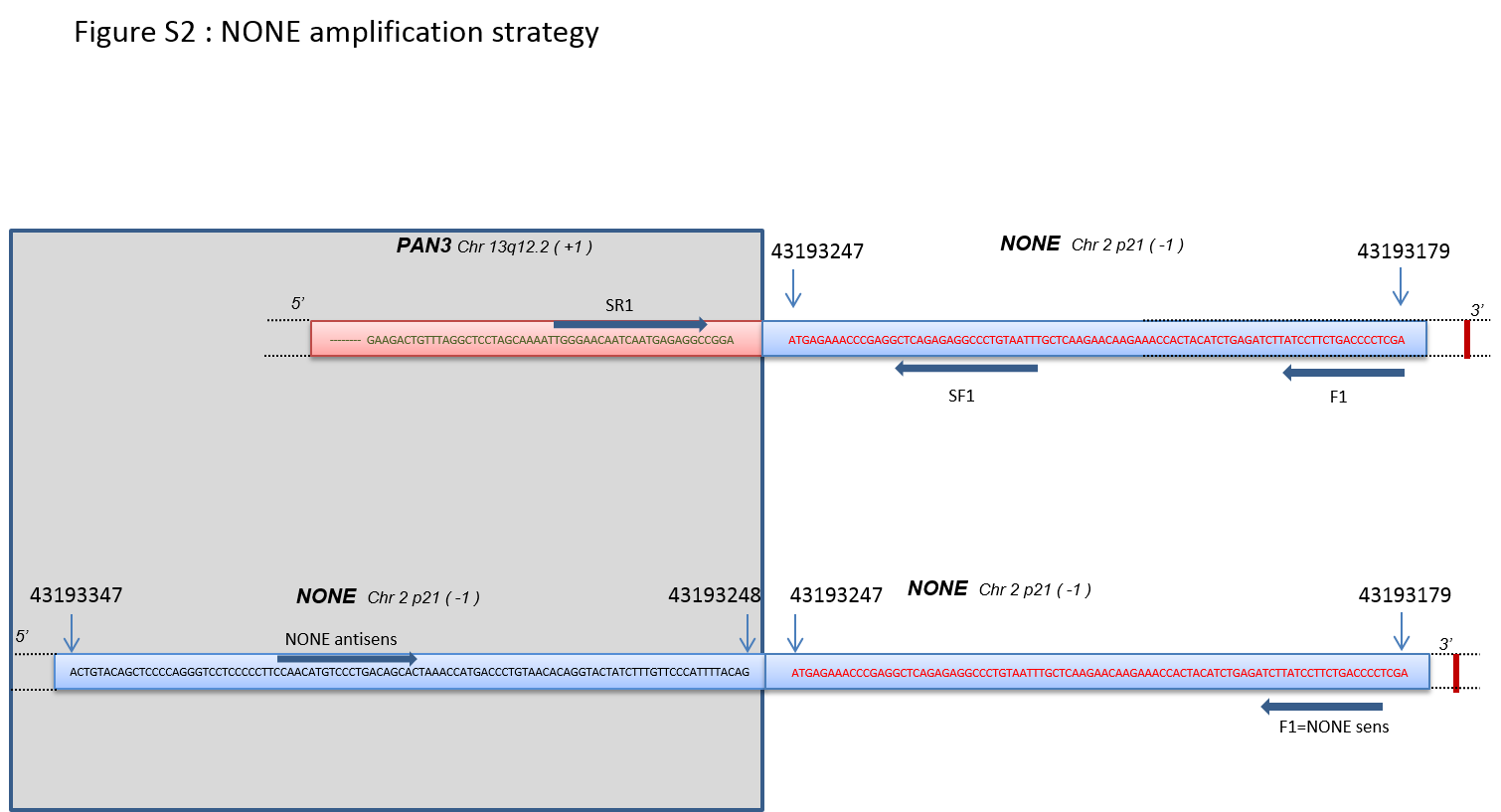

Supplement: Supplementary file 2 [file f1000research-6-14532-s0001.tgz › da03f903-cbb5-4c26-bcdf-185fc885f30f.tif]

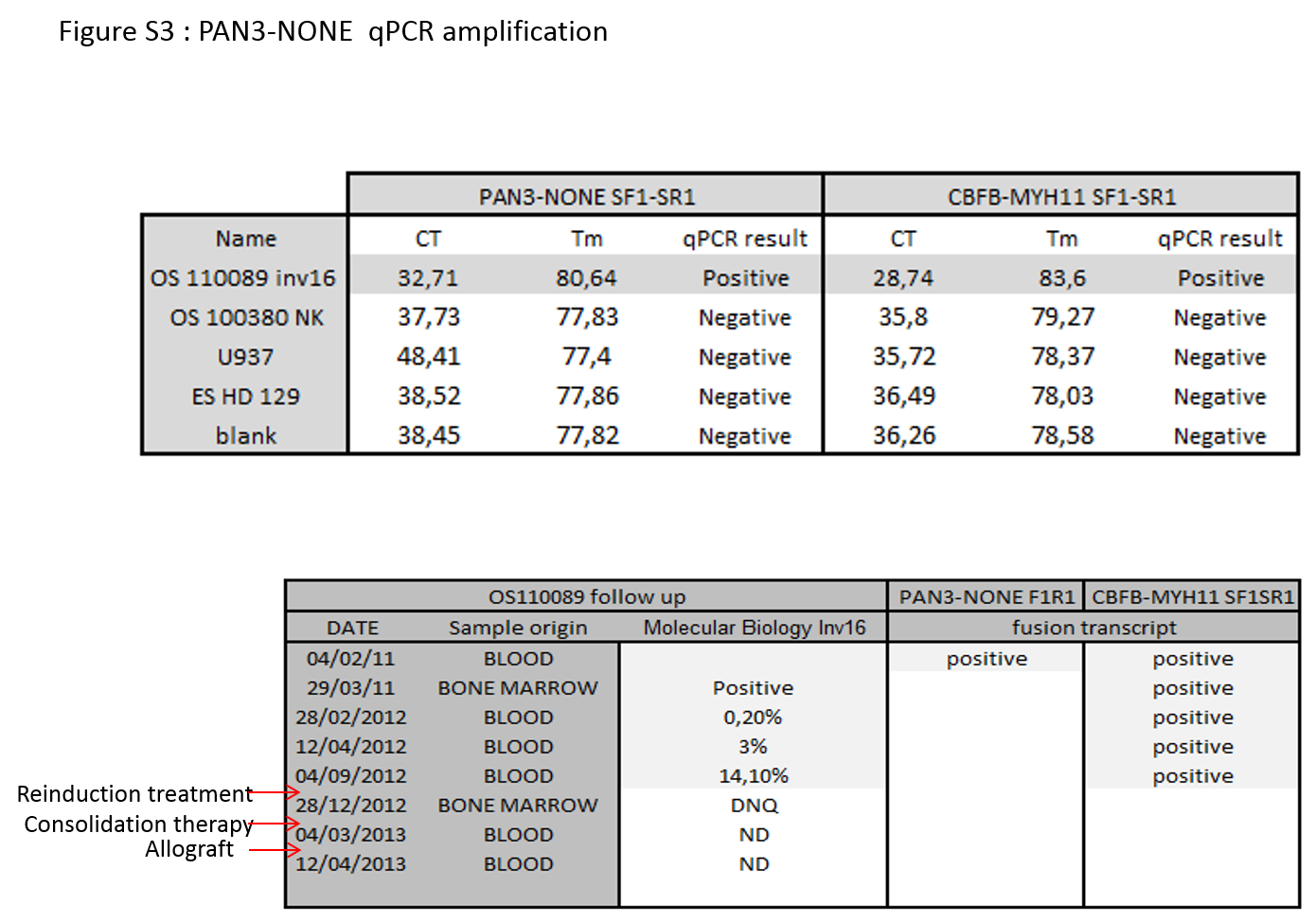

Supplement: Supplementary file 3 [file f1000research-6-14532-s0002.tgz › 967b8e70-931d-44c6-9bf8-6872b74c2358.tif]

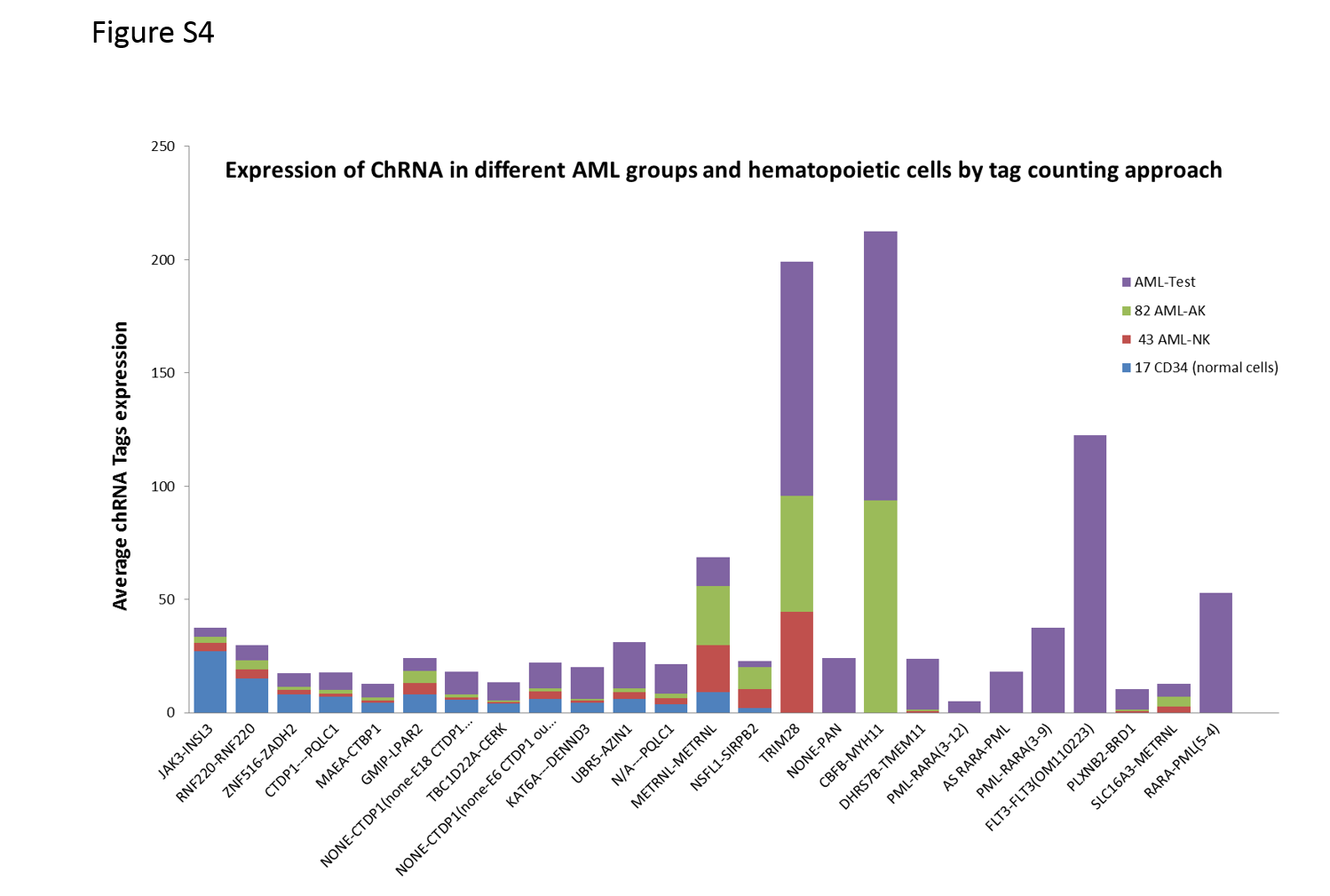

Supplement: Supplementary file 4 [file f1000research-6-14532-s0003.tgz › b8ff0983-0fc9-4135-9718-547e9326b1b8.tif]

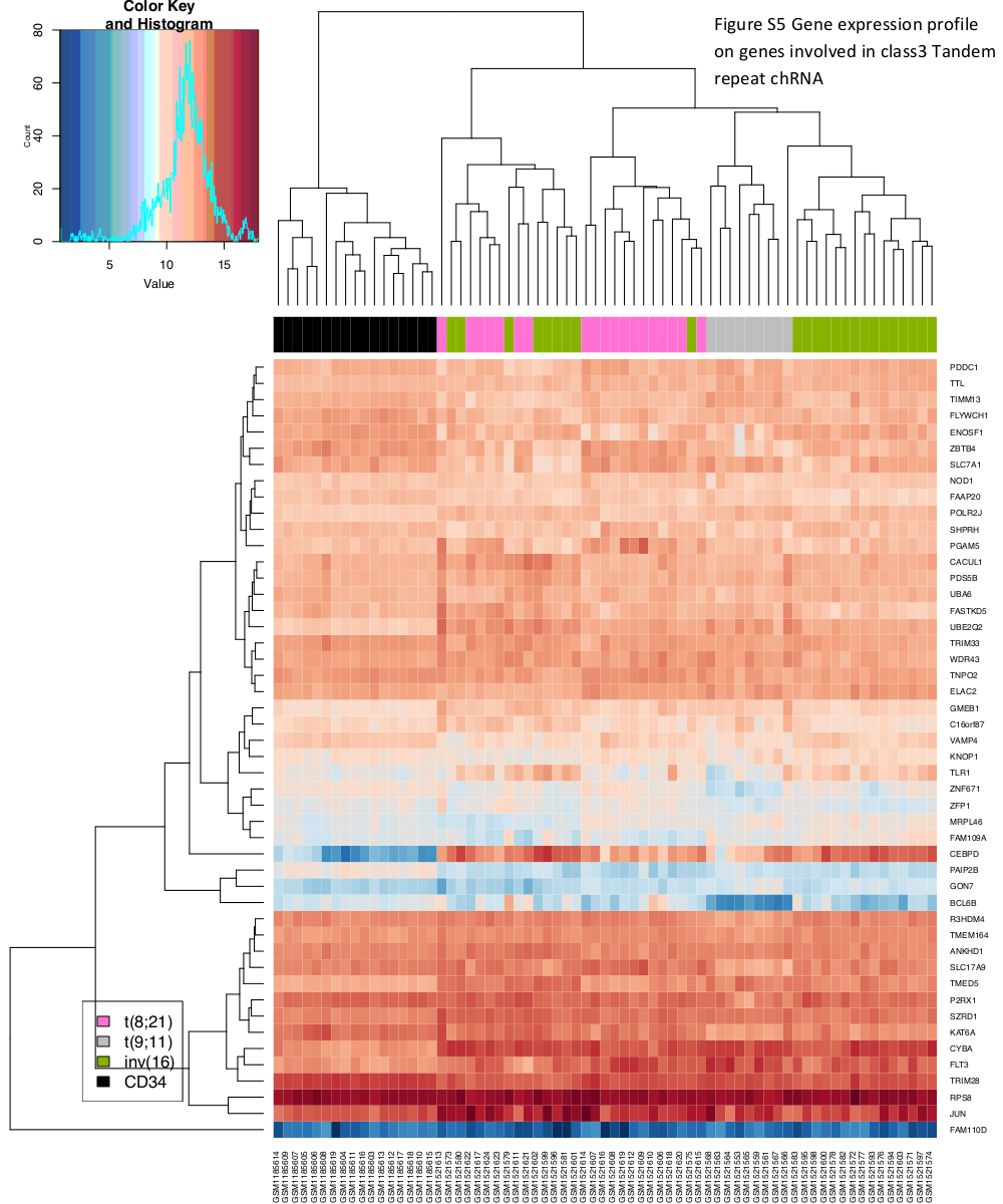

Supplement: Supplementary file 5 [file f1000research-6-14532-s0004.tgz › 184bc463-3c23-437c-918a-384da164e5fc.tif]
