# Supplementary material for: New chimeric RNAs in acute myeloid leukemia
Source: F1000Res. 2017 Dec 19;6:ISCB Comm J-1302. Originally published 2017 Aug 2. [Version 2] doi: 10.12688/f1000research.11352.2 (PMC5861515; doi:10.12688/f1000research.11352.2)
Supplement: Peer review history

# Peer review: Referee response for version 2

**DOI:** 10.5256/f1000research.14532.r29147

**Published:** 2018-03-16

**Authors:** Charles Gawad

**Affiliations:** 1 Department of Computational Biology, St. Jude Children's Research Hospital, Memphis, TN, USA

I am satisfied with the authors responses and feel the report is now suitable for indexing.

I have read this submission. I believe that I have an appropriate level of expertise to confirm that it is of an acceptable scientific standard.

# Peer review: Referee response for version 1

**DOI:** 10.5256/f1000research.12254.r25306

**Published:** 2017-10-03

**Authors:** Charles Gawad

**Affiliations:** 1 Department of Computational Biology, St. Jude Children's Research Hospital, Memphis, TN, USA

In this report, Ruffle et al. present a new approach for identifying novel chimeric transcripts using CracTools. They classify the transcripts into four categories: 1)  different chromosomes (interchromosomal translocations), 2) co-linear but from different genes (putative transcriptional read through or complex intrachromosomal structural variation), 3) exons from same chromosome but are not in the expected order based on the reference (tandem duplication, complex intrachromosomal rearrangement), or 4) exons on same chromosome but different strands. The authors then go on to validate a subset of putative new chimeric transcripts using RT-PCR and FISH. These types of studies have been previously performed. One new aspect of this study is the stranded library preparation which allows for the identification of the class 4 chimeras. In addition, they tried to minimize the bias in their analysis pipeline by not relying on a reference genome, which enabled the discovery of new transcripts. Overall, their approach provides a validated new strategy for identifying novel transcripts in RNA-seq data.

Major Concerns

1. The authors do not discuss circular RNA, which are likely to make up a large portion of their class 3 chimeras as found in many recent studies. The low numbers of class 3 chimeras also raises concerns about the sensitivity of the approach, as most recent studies have found thousands of circular RNA isoforms per sample. The total RNA underwent RT with random primers, which would retain circular RNA. It is not clear if there was a polyA-selection step after that point or if the total RNA was ribosomal depleted. If it was the former, the circular transcripts would not be present.
2. If the RNA was not polyA-selected, the authors should specifically discuss the PML-RARA circular transcripts recently discovered in acute promyelocytic leukemia. I am not aware of any independent validation of that work.

Minor Concerns

1. The authors should read the manuscript closely for typos. For example in the AML samples and cells lines section there are commas where there should be periods, in the FISH methods section there are degree signs instead of percent, and two paragraphs before the discussion there is MDR instead of MRD.
2. The authors should include the kits used for ribosomal RNA-depletion/polyA-selection, as well as stranded library preparation.
3. What mechanisms do the authors have in mind for a structural variant and/or alternative splicing event that would result in class 4 chimeras, as they would not occur as a result of transcriptional read through of the same strand?
4. The resolution is too low for Figure 5A.

I have read this submission. I believe that I have an appropriate level of expertise to confirm that it is of an acceptable scientific standard, however I have significant reservations, as outlined above.

## References

[1] SalzmanJGawadCWangPLLacayoNBrownPO: Circular RNAs are the predominant transcript isoform from hundreds of human genes in diverse cell types. *PLoS One*.2012;7(2) : 10.1371/journal.pone.0030733 e30733 10.1371/journal.pone.0030733 22319583PMC3270023

[2] JeckWRSorrentinoJAWangKSlevinMKBurdCELiuJMarzluffWFSharplessNE: Circular RNAs are abundant, conserved, and associated with ALU repeats. *RNA*.2013;19(2) : 10.1261/rna.035667.112 141-57 10.1261/rna.035667.112 23249747PMC3543092

[3] GuarnerioJBezziMJeongJCPaffenholzSVBerryKNaldiniMMLo-CocoFTayYBeckAHPandolfiPP: Oncogenic Role of Fusion-circRNAs Derived from Cancer-Associated Chromosomal Translocations. *Cell*.2016;165(2) : 10.1016/j.cell.2016.03.020 289-302 10.1016/j.cell.2016.03.020 27040497

# Response

**Published:** 2017-12-05

**Authors:** Therese Commes

**Affiliations:** Universite de Montpellier, France

We thank the referee for his careful reading of our manuscript and for his remarks which led us to clarify the protocol used in this work.

**Major Concerns **

- *The authors do not discuss circular RNA, which are likely to make up a large portion of their class 3 chimeras as found in many recent studies. The low numbers of class 3 chimeras also raises concerns about the sensitivity of the approach, as most recent studies have found thousands of circular RNA isoforms per sample. The total RNA underwent RT with random primers, which would retain circular RNA. It is not clear if there was a polyA-selection step after that point or if the total RNA was ribosomal depleted. If it was the former, the circular transcripts would not be present*.

 

Response: The remark is relevant and we agree with the referee that it was not clearly stated that we performed polyA selection for the RNAseq experiment. This point is only described in online data availability (https://www.ebi.ac.uk/arrayexpress/experiments/E-MTAB-5767/) and doesn’t appear explicitly in mat&methods section. We added the following comment on page 4: “ *The RNAseq was performed using polyA-selection with the TruSeq RNA Lib-Prep Kit (Illumina) adjusted with GATC specific procedure for strand specificity”*.

 We also agree with the referee that class3 fusion transcripts could arise from circular RNA (circRNA) when performing ribosomal depleted RNA-seq. However, as our polyA+ RNA-seq study doesn’t enable the identification of this RNA subtype, we do not discuss about circRNA in the manuscript. Concerning class3 chimeric RNA, our pipeline detects a great number of candidates before applying specific filters. This category, as well as the class2, is the most represented in all datasets we analyzed. As described in mat&methods section, we used stringent criteria to minimize bias and greatly reduced the number of candidates.

 For example, from a typical AML stranded and paired- end RNA-seq experiment (50 Millions reads; 100pb length), starting from the raw data (CRAC and CracTools process) we extracted 1406 chRNAs including 35% of class3chRNA reduced by 16 fold with the filtering process (26 class3chRNA).

  

- *If the RNA was not polyA-selected, the authors should specifically discuss the PML-RARA circular transcripts recently discovered in acute promyelocytic leukemia. I am not aware of any independent validation of that work.*

 

Response: As we choose polyA RNA-seq, we can only characterize new linear fusion transcripts. From ribo-depleted RNAseq data analysis, the characterization of circRNA arising from fusion gene requires the identification of linear junction (lin-J) and circular junction (circ-J) described as spliced and back spliced junction respectively by Guarnerio et al ( *Oncogenic Role of Fusion-circRNAs Derived from Cancer-Associated Chromosomal Translocations, 2016*; *Cell* 165 (2): 289-302 *). *The linear and circular fusion transcripts share the same linear junction.

 The new (3-12) PML-RARA junction we discover corresponds necessarily to a linear junction because of its fusion junction sequence (see figure at https://bio2m.montp.inserm.fr/papers/AMLchimera2017). Moreover, in our polyA+ dataset it surely corresponds to a linear transcript. It is most probably an alternative splicing product transcribed from the PML-RARA bcr3 translocation since it coexists with the well-known (3-9) PML-RARA bcr3 transcript. However we could not exclude in the biological sample the presence of circRNA emerging from the PML-RARA fusion gene. However polyA RNAseq protocol doesn’t enable to reveal them.

 To answer to the referee remark, we performed complementary experiments and analyzed the Guarnerio dataset ( *BioProjectID: PRJNA315254*) with our pipeline to detect fusion junctions (lin-J and Circ-J) specific to *circRNA* arising from the PML-RARA translocated genes. We found 3 PML-RARA and 1 RARA-PML linear fusion junctions but we did not detect the circRNA ones. We performed a tag search approach specific to the PML-RARA circ-junction (F-circ1) described in Fig S1B supplemental information of Guarnerio et al manuscript. We did not find it in their fastQ files. To conclude we were unable to detect PML-RARA cirRNA in Guarnerio J dataset but we confirmed the data recently described by You, X. and Conrad, T. OF  with the  specific cirRNA Acfs pipeline ( *Acfs: accurate circRNA identification and quantification from RNA-Seq data, Scientific Report 6,38820; doi: 10.1038/srep38820; 2016*). The circRNA search for low abundance will certainly require more deepness in RNAseq.

**Minor Concerns **

- *The authors should read the manuscript closely for typos. For example in the AML samples and cells lines section there are commas where there should be periods, in the FISH methods section there are degree signs instead of percent, and two paragraphs before the discussion there is MDR instead of MRD.*

Response: We read carefully the manuscript and corrected the typos.

  

- *The authors should include the kits used for ribosomal RNA-depletion/polyA-selection, as well as stranded library preparation.*

Response: We added the following comment on page 4: “ *The RNAseq was performed using polyA-selection with the TruSeq RNA Lib-Prep Kit (Illumina, San Diego, CA) adjusted with GATC specific procedure for strand specificity”*.

  

- *What mechanisms do the authors have in mind for a structural variant and/or alternative splicing event that would result in class 4 chimeras, as they would not occur as a result of transcriptional read through of the same strand ?*

Response: Two mechanisms would result in class 4 chimeras. The first one could involve chromosomic duplication and inversion as described in Newman et al *(* *Next-generation sequencing of duplication CNVs reveals that most are tandem and some create fusion genes at breakpoints,* *Am J Hum Genet.* *2015, Feb 5; 96(2):208-20. doi:10.1016/j.ajhg. 2014.12.017. Epub 2015 Jan 29* *). *The second one could involve splicing events as described by Gingeras et al ( *Implications of chimaeric non-co-linear transcripts,* *Nature.* *2009 Sep 10; 461(7261):206-11doi: 10.1038/nature08452.*)

  

- *The resolution is too low for Figure 5A.*

Response: We changed the resolution of the figure 5A, increasing the font size.

# Peer review: Referee response for version 1

**DOI:** 10.5256/f1000research.12254.r25101

**Published:** 2017-08-29

**Authors:** Hui Li

**Affiliations:** 1 Department of Pathology, School of Medicine, University of Virginia, Charlottesville, VA, USA

Ruffle et al., performed RNA-Seq on three AML patients, and identified a number of new chimeric RNAs. They grouped them into reasonable categories, and discussed their generating mechanisms. For some, they performed tag search in larger sets of RNA-Seq data of AML and CD34+ HSCs. In general, the analysis is solid. Multiple levels of evidence were provided for some fusion RNAs (for instance FISH for a Class I fusion). The conclusion is justified.

 No major issues were noted.

 Minor suggestions:

1. It would be nice to perform the same RNA-Seq pipeline analysis on bigger datasets, such as the data from LEUCEGENE.
2. It is well known that different software tools behave differently and false positive/negative is a big issue. Would be nice to use another independent software for crosschecking.
3. The METRNL001 Class 3 chimera may be a product of backsplicing if the sequencing protocol is not restricted to polyA RNAs.

I have read this submission. I believe that I have an appropriate level of expertise to confirm that it is of an acceptable scientific standard.

# Response

**Published:** 2017-11-17

**Authors:** Therese Commes

**Affiliations:** Universite de Montpellier, France

**Response to referee Report ( Hui LI, Department of Pathology, School of Medicine, University of Virginia, Charlottesville, VA, USA)**

*1. It would be nice to perform the same RNAseq pipeline analysis on bigger datasets, such as the data from leucegene.*

*2. It is well known that different software tools behave differently and false positive /negative is a big issue. Would be nice to use another independent software for crosschecking.*

*3. The METRNL001 class3 chimera may be a product of backsplicing if the sequencing protocol is not restricted to polyA RNAs*

We want to thank the referee for taking time to read the manuscript and provide constructive feedback.

1. The first remark concerning LEUCEGENE analysis is relevant as this cohort is a unique dataset of 450 RNA-seq from AML patients and normal hematopoietic cells. This cohort includes diverse types of AML and clinical data with a deepness of 100-300x10 ^6^ reads/per sample. However, our first aim in the present manuscript was to test our pipeline and to propose a new way to analyze, select and classify chRNA. We thus choose to analyze a small dataset and used the Leucegene cohort only to explore specific expression of the new chRNAs (revealed with our pipeline). This Expression with a tag search approach was based on their recurrence, tumor, subgroup, or patient-specific expression. The complete leucegene chRNA research represents a big task and constitutes a full-fledged work, we are now performing our RNAseq pipeline on this dataset. In this new task, we will check at a large scale the pipeline performance, in term of memory computing and time consuming, and also validate its capacity to detect all the well-known AML fusion genes. We then should be able to discover new chimeric RNA in AML.

 2. We totally agree with the referee and are well aware of potential bias generated with different pipelines. Our group is particularly concerned about software comparison for chimeric RNA detection *. *CRAC was developed in the Lab and compared with other software before we choose it for this study ( *Philippe et al, 2013, Genome Biology; an integrated approach to the analysis of RNA-seq reads* genome Biology) and we recently published in September 2017 a benchmark method dealing with this topic *(Audoux et al, 2017, BMC Bioinformatics; SimBA: A methodology and tools for evaluating the performance of RNA-Seq bioinformatic pipelines)*. CRAC tools has been developed in the Lab in response to biologists requests to better characterize, filter and classify predicted fusion transcripts. Moreover, we recently compared the pipeline CRAC and CRACtools with Fusioncatcher in our AML dataset, as it provides interesting and complementary annotation (overlapping databases, protein impact) but the process is time consuming and the filtering step is more stringent, many of the new fusion transcripts we validated are ruled out.

Whatever the bioinformatics pipeline used, bias will subsist. In order to improve chRNA discovery, we undertook to couple the bioinformatics approach with biological information and propose a tag search approach targeting the chimeric junctions in normal and tumoral large data set. We demonstrate that this complementary approach works well and will help the biologist to determine the biological relevance of chimeric events.

3. Since we use polyA+ RNA to perform RNAseq, the METRNL001 class3 chimera, which involves a single exon (end of exon 2 METRNL transcribed before the start of the same exon) more probably corresponds to a linear polyA+ transcript involving a short repeat sequence of 5’ part of exon 2 as described in Fig4 rather to a circularization of a single exon formed by backsplicing.
